# Lignin Reinforcement in Polybutylene Succinate Copolymers

**DOI:** 10.3390/polym17020194

**Published:** 2025-01-14

**Authors:** Nnaemeka Ewurum, Armando G. McDonald

**Affiliations:** Department of Forest and Fire Sciences, University of Idaho, Moscow, ID 83844-1132, USA; newurum@uidaho.edu

**Keywords:** lignin reinforcement, polybutylene succinate (PBS), biopolymers, mechanical properties, thermal stability, crosslinking, renewable materials, biodegradable composites

## Abstract

This study investigated the valorization of industrial lignin for producing biodegradable polybutylene succinate (PBS)–lignin copolymers. PBS was blended with varying lignin contents (0–45 wt. %) and crosslinked/grafted using dicumyl peroxide (DCP). The preparation of the copolymers by reactive extrusion was successful, with mechanical, thermal, and morphological properties comprehensively analyzed. Lignin addition decreased tensile strength but improved stiffness (modulus) and thermal stability. Crosslinking with DCP improved the interfacial adhesion between PBS and lignin, resulting in better flexural performance at moderate lignin levels. Differential scanning calorimetry showed that lignin initially improved the crystallization temperature, but hindered it at higher concentrations due to its rigid, aromatic structure. Scanning electron microscopy analysis showed poor interfacial adhesion in PBS–lignin blends, but the surface morphology improved in crosslinked PBS–lignin copolymers, with less phase separation observed. An optimal lignin concentration appeared to depend on the property of interest. While 30% lignin provided the best improvement in flexural strength, 20% lignin offered a more balanced enhancement for most properties without the severe reduction in tensile strength observed at higher lignin contents.

## 1. Introduction

Bioplastics, which include bio-sourced and biodegradable plastics, have recently gained a lot of attention because they do not come with the level of environmental pollution problems associated with conventional petroleum-based plastics [1,2]. These materials are both renewable and biodegradable, and as a result, they make a more sustainable alternative to conventional plastics, especially by reducing greenhouse gas emissions and conserving what is left of the mostly nonrenewable petroleum resources [3]. Studies have shown that, in addition to decreasing the carbon footprint of plastic production, the use of bioplastics will significantly reduce the dependence on fossil fuel, therefore supporting the global effort towards sustainability and the conservation of the environment [4,5,6]. In addition, bioplastics have the potential to decompose more easily than conventional plastic, which would address the challenges of pollution in oceans and landfills. This factor is important considering the increasing global accumulation of plastic waste [7]. Bioplastics are versatile in their applications, they are feasible for use in mass manufacturing and they show potential for low-cost production in the long run; this creates a need for research and development in the field to maximize the industrial potential of these materials [8]. In addition, bioplastics play an important part in promoting circular economic practices by encouraging recycling methods and improving the sustainability of products.

Lignin is a natural aromatic polymer found in plant cell walls. It is considered as one of the resources that could aid in enhancing the sustainability of bioplastics [9]. Historically, lignin was considered a low-value waste product of the paper pulping industrial process. However, its abundance and unique beneficial properties have drawn the attention of researchers [10]. By using lignin as a component in bioplastic products, its properties can be maximized, which would further enhance the sustainability of these products. Some of the beneficial properties of lignin include biodegradability, thermal stability, low density, good mechanical properties, etc., thereby making it a viable option for copolymerization with biopolymers such as polybutylene succinate (PBS), polyhydroxybutyrate-co-valerate (PHBV), polylactic acid (PLA), etc. [11]. In addition, lignin has been found to possess antioxidant and antimicrobial properties that make it promising for use in applications in the packaging and healthcare industries [12,13]. Lignin can be extracted from various biomass sources, including softwood and hardwood, which makes it highly renewable and sustainable [14]. Moreover, the structural diversity of lignin provides opportunities to tailor the properties of derived bioplastics [15]. Lignin has been incorporated into PHBV by grafting [16] and PBS by a condensation reaction [17], the properties of which vary based on lignin content.

Polybutylene succinate is an aliphatic polyester that has been gaining an increasing amount of attention because its precursors are renewable, and it exhibits properties that are comparable to conventional plastics such as polyethylene [18]. The precursors of PBS are succinic acid and 1,4-butanediol, and these can be sourced from renewable resources. Succinic acid is a platform chemical that can be produced through various sustainable methods, including the fermentation of biomass. For instance, *Actinobacillus succinogenes* has been shown to successfully convert glucose and other carbohydrates obtained from renewable sources into succinic acid [19]. Butanediol can be derived from renewable sources through various biological and chemical processes. One of the most promising methods is fermentation, whereby specific microorganisms convert sugars from biomass into butanediol. For instance, engineered strains of bacteria, such as *Escherichia coli*, have been successfully used to ferment xylose to produce butanediol [20]. PBS is not only biodegradable and sustainably sourced; it also possesses good mechanical strength, flexibility, and thermal stability, comparable to other popular bioplastics like PLA and PHA (polyhydroxyalkanoates). These properties make PBS ideal for various applications such as packaging, agriculture, textiles, construction, and even biomedical applications [21,22,23,24]. While PBS is biodegradable and sourced from renewable resources, its high production costs, driven by expensive precursors, remain a barrier to its widespread adoption [25]. Incorporating lignin, a low-cost by-product of the paper industry, offers a promising method to reduce production costs while retaining biodegradability and enhancing specific material properties, such as thermal stability and stiffness. This study focuses on optimizing the lignin content in PBS–lignin copolymers in order to find a balance between cost reduction and material performance.

Unmodified lignin can be incompatible with aliphatic polyesters such as PBS, due to lignin’s heterogenous structure and differences in polarity, which result in poor mechanical properties and a lack of uniformity in the composite material [26]. Crosslinking agents like dicumyl peroxide (DCP) are often used to address these compatibility issues [16,27,28]. DCP helps to create copolymers and composites by chemically linking the lignin to the PBS, improving the overall interaction between the two materials. This crosslinking process improves the mechanical properties of the composite and helps to stabilize the structure [28,29].

The addition of lignin improves certain properties of PBS, such as thermal stability and flexural modulus, but can negatively affect other key properties, particularly tensile strength [30]. This study seeks to determine the optimal lignin content that maximizes these improvements while minimizing the loss in mechanical performance. The results will inform a follow-up study that will investigate the potential of incorporating lignocellulosic reinforcements like hemp fiber to offset these performance trade-offs and further optimize the material properties. This study will prepare PBS–lignin copolymers by reactive extrusion, with varying concentrations of lignin, followed by the comprehensive characterization of these copolymers. In addition, this study will also investigate the potential effects of DCP addition on copolymer properties.

## 2. Materials and Methods

### 2.1. Materials

The polybutylene succinate (PBS) pellets (BioPBS™ FZ91PM/FZ91PB, Mitsubishi Chemical Group, Bellevue, OH, USA) have a density of 1.26 g/cm^3^ and a melt flow ratio (MFR) of 5 g/10 min, measured at 190 °C under a load of 2.16 kg. Prior to use, the PBS pellets were ground using a Thomas–Wiley mill and passed through a 2 mm screen to achieve the desired particle size for blending with lignin for extrusion. Dicumyl peroxide (DCP) with 99% purity, obtained from Acros Organics (Morris Plains, NJ, USA), served as the crosslinking agent in this study. Softwood kraft lignin (Indulin AT, Westvaco, SC, USA) was supplied by Westvaco.

### 2.2. Material Characterization

The particle size distribution of kraft lignin was analyzed using wet dispersion on a Bettersizer 2600 (Costa Mesa, CA, USA). The density of kraft lignin (2 g) was measured by gas pycnometry on an ultra-pycnometer 1000 (Quantachrome, Boynton Beach, FL, USA) using Nitrogen (Oxarc Gases, Lewiston, ID, USA).

### 2.3. Copolymer Preparation

In this study, lignin was incorporated into PBS copolymers at four different concentrations: 10%, 20%, 30%, and 45% by weight. The lignin was added as a percentage of the total polymer mass to evaluate its effects on the material properties. The trial plan involved preparing samples with increasing lignin contents to assess the influence of lignin on the mechanical, thermal, and structural properties of the resulting copolymers. Dicumyl peroxide (DCP) was dissolved in acetone and thoroughly mixed with milled PBS to ensure the uniform dispersal of the DCP on the PBS surface. The mixture was left to dry for 24 h in a fume hood, and then further dried in a vacuum oven at 80 °C for 12 h. Two series of PBS–lignin (simple blends and reaction-extruded copolymers with DCP) materials were prepared with varying concentrations of kraft lignin (0–45 wt. %). The reaction extrusion co-polymers contained 0.25 wt. % DCP added to PBS. The various formulations (50 g) were separately fed into a single-screw extruder (Wellzoom EZ3DX EASY 3D PRINTER, Shenzhen Mistar Technology Co., Ltd., Shenzhen, China; 10 mm Ø) and extruded at 140 °C to produce 3 mm Ø strands. These strands were subsequently cut into pellets. The resulting pellets (30 g) were molded into discs using a 75 mm Ø pellet die in a hot press (PHI 30-ton hydraulic press, South El Monte, CA, USA) at 120 °C, to produce discs with a thickness of 3.1 mm, which were then cut into rectangular strips for testing. Portions of the copolymers were compounded using a Dynisco Lab Mixer Molder/Extruder (LMM, Franklin, MA, USA) at 100 rpm for 10 min at 140 °C and then injection-molded into dog bone specimens and disc specimens. The extruded materials were labeled as Rxx for the reaction-extruded (with DCP) materials, where xx represents the percentage of lignin (e.g., R20 for 20% lignin). For PBS–lignin blends, the samples were labeled Bxx, where xx similarly represents the lignin concentration (e.g., B20 for 20% lignin).

### 2.4. Gel Fraction

The gel fraction was determined on hot-pressed DCP cross-linked PBS–lignin copolymers that were Soxhlet-extracted with CHCl_3_ for 48 h to remove the soluble “sol” fraction. The undissolved “gel” fraction was collected and dried in the vacuum oven for 48 h. The yields were calculated as follows:

Gel fraction (%) = (W_gel_/W_0_) × 100 (1)

### 2.5. Scanning Electron Microscopy (SEM)

SEM was performed on gold-coated fractured surface samples on a Zeiss Supra 35 VP (Dublin, CA, USA) equipped with a secondary electron detector (SE2) at 10 KV.

### 2.6. Rheology

The rheological behaviors of the PBS–lignin blends and cross-linked copolymers (25 mm Ø × 2.0 mm) were studied using a parallel plate rheometer (Bohlin CVO 100 NF, East Brunswick, NJ, USA) from 0.01 to 100 Hz (0.0628–628 rad·s^−1^), at 0.025% strain and 140 °C. Data were analyzed (power-law models) using the Bohlin rheology v6.51.0.3 and Microsoft Excel software. Complex viscosity (η*), elastic modulus (G′), tan δ, and viscous modulus (G″) were measured. A modified power–law model was applied to the rheological data to quantify the shear thinning characteristics, as expressed in Equation (2),

|η* (ω)| = 𝐾(ω)^𝑛−1^ (2)

where |η* (ω)| is the complex viscosity as a function of angular frequency ω, *K* is the consistency index, and *n* is the flow behavior index.

### 2.7. Fourier Transform Infrared Spectroscopy (FTIR)

The FTIR spectra of copolymer samples were obtained using a Nicolet-iS5 spectrometer (Thermo-Scientific, Madison, WI, USA) over 64 scans, with an attenuated total reflection (ATR) probe (ZnSe crystal). The spectra were baseline-corrected and averaged using the Omnic v9.8 software.

### 2.8. Thermal Analysis

Dynamical mechanical analysis (DMA) was performed, in triplicate, on rectangular bar specimens (3 × 5 × 20 mm^3^) using a 3-point bending fixture with a 15 mm span on a Perkin Elmer DMA-7 instrument (Shelton, CT, USA) at a frequency of 1 Hz, with 0.2% strain and from −50 to 120 °C at 3 °C/min. The data were analyzed with Pyris v13.3 software. The melting temperature (T_m_), crystallization temperature (T_c_), and degree of crystallization (*X*_c_) of the extruded copolymers (10 mg) were determined by differential scanning calorimetry (DSC) on a Perkin Elmer DSC-7 instrument from 30 to 150 °C at 10 °C/min. The samples were ramped to 150 °C at 10 °C/min and then cooled to 30 °C at −10 °C/min, which was followed by a second ramp to 150 °C at 10 °C/min. The crystallinity of the polymers (*X*_c_) was calculated using Equation (3),

𝑋𝐶 = Δ𝐻_m_/𝑓Δ𝐻_0_ × 100 (3)

where ΔH_m_ is the melting enthalpy derived from the area under the peak, *f* is the weight fraction of the polymer in the formulation and ΔH_0_ (110.3 J/g) is the enthalpy of fusion of 100% crystalline PBS [31].

The thermal stabilities of kraft lignin, PBS, and copolymers (5 mg) were determined by TGA using a PerkinElmer TGA 7 instrument (Shelton, CT, USA) from 30 to 800 °C at 20 °C/min under nitrogen (30 mL/min). The TGA was calibrated with alumel, perkalloy, nickel, and iron standards. The data were analyzed using Pyris v13.3.1 software.

### 2.9. Mechanical Properties

The tensile tests were performed on injection-molded microtensile (dog-bone) specimens (10 replicates) according to the ASTM Standard D1708 [32] using an Instron 5500R-1132 universal test machine, equipped with Bluehill v3.3 software, with a crosshead speed of 1 mm/min and strain measured using an extensometer (model 3542, Epsilon Technology Corp., Jackson, WY, USA). Three-point flexural tests (8 replicates) were performed using a 2.5 kN capacity Mecmesin MultiTest-dV (PPT Group, Slinfold, UK) equipped with VectorPro Lite V6.1.0.0 Software at a crosshead test speed of 1.1 mm/min according to ASTM standard D790 [33].

### 2.10. Data Analysis

Statistical analyses were conducted using Tukey’s pairwise comparisons and *t*-tests to evaluate differences between formulations. The results were considered statistically significant at a confidence level of 95% (*p* < 0.05).

## 3. Results

### 3.1. Extrusion of PBS–Lignin Formulations

PBS was milled to a granular powder, which could be blended uniformly with kraft lignin powder (average particle size of 166 µm) prior to extrusion. PBS–lignin blends and DCP cross-linked PBS–lignin materials (0–45% lignin) were prepared by extrusion. The simple blends would be soluble in CHCl_3_, while the DCP cross-linked formulations would form a three-dimensional networked gel. The gel fraction results (Table 1) show that cross-linking increased with lignin content. At lower lignin levels (R10 and R20), the gel fraction was low, about 4–5%, indicating minimal crosslinking. However, the gel fraction increased significantly with increasing lignin content, reaching 26.2% for R45. This suggests that lignin facilitates crosslinking/grafting by providing additional sites for hydrogen abstraction and radical formation. Although higher lignin levels improve gel formation, the DCP content (0.25%) used in this study may have limited the extent of crosslinking. Luo et al. [16] found that the highest gel content occurred with 0.5 wt. % DCP, indicating that increasing the DCP content could potentially further enhance crosslinking. Ma et al. [34] also observed that insufficient amounts of crosslinking agents led to low gel content in lignin-based polyurethanes.

The density values of PBS–lignin formulations show that lignin addition had only a very slight effect on density (Table 1). The density of neat PBS, B0, was 1.244 g/cm^3^, and decreased slightly with lignin content to 1.239 g/cm^3^ in B45. For cross-linked PBS–lignin copolymer R45, the density was 1.246 g/cm^3^. This indicates that lignin can be incorporated into PBS without significantly altering its density, allowing for reinforcement benefits like improved stiffness or thermal stability.

### 3.2. FTIR Spectroscopy (FTIR)

**Figure 1:**
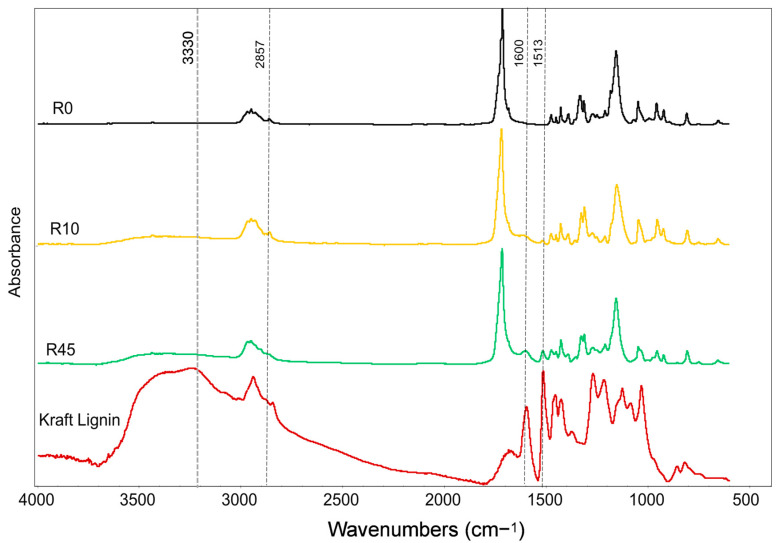
FTIR spectra of PBS, lignin, and DCP cross-linked PBS–lignin copolymers.

The FTIR spectra of the cross-linked samples (R0, R10, R45) were analyzed in comparison to pure kraft lignin and neat PBS, focusing on changes as the lignin content increases (Figure 1). The bands at 1157 cm^−1^, 1718 cm^−1^, and 1333 cm^−1^, corresponding to the C–O stretching, C=O stretching, and symmetric C–O stretching in PBS, remained unchanged. This suggests that the ester backbone of PBS retains its structural integrity during copolymerization with lignin [35]. However, the broad band around 3000–3500 cm^−1^, associated with O–H stretching vibrations, showed an increased intensity with higher lignin content, likely due to the enhanced hydrogen bonding from lignin’s hydroxyl groups. Additionally, new bands emerged at 1600 cm^−1^ and 1513 cm^−1^, attributed to lignin’s aromatic skeletal vibrations. These bands became more prominent as lignin content increased, indicating a greater integration of lignin’s aromatic structure into the copolymer matrix [36]. A band at 1269 cm^−1^ was assigned to guaiacyl (ring breathing and carbonyl stretching) units of lignin. The band at 2857 cm^−1^, corresponding to C–H stretching in aliphatic chains, decreased with lignin content, possibly reflecting lignin’s influence in reducing the contribution of these aliphatic groups [37].

### 3.3. Rheology of PBS–Lignin Melts

**Figure 2:**
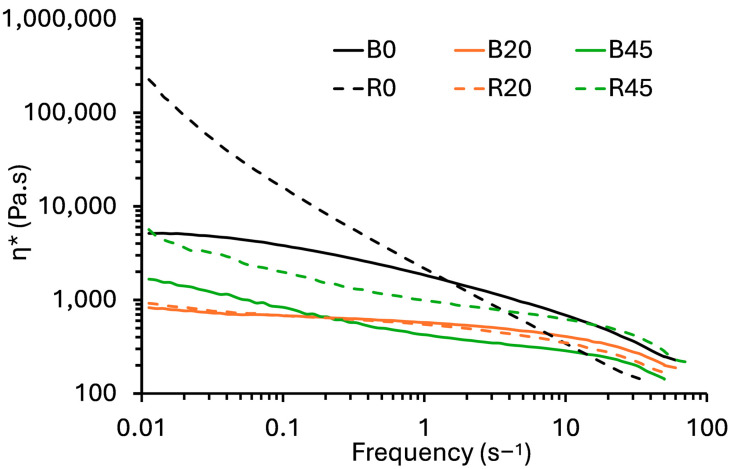
Flow curves (η* versus frequency) of blended and crosslinked PBS–lignin copolymers.

The flow characteristics (complex viscosity (*η*∗) versus frequency) of PBS, PBS–lignin blends and cross-linked PBS–lignin polymer melts were evaluated by dynamic rheology at 140 °C (Figure 2). For the PBS–lignin blends (samples B0 to B45), *η*∗ generally decreased with lignin content. For comparative purposes, the *η*∗ at 1 Hz (and 30 Hz) were used, and the data are summarized in Table 2. For example, *η*∗ for PBS (B0) was 1.81 kPa.s and dropped to 0.422 kPa.s for B45. Lignin acts as a filler that disrupts the polymer matrix, reducing chain entanglements and improving flow under shear stress. This decrease in *η*∗ with increasing lignin content is consistent with the behavior observed in other lignin-filled polymers [38,39]. The PBS–lignin extruded with the DCP series (R0–R45) generally (except for R10 and R20) showed higher *η*∗ values at 1 Hz than the blended material at the same lignin content. For example, *η*∗ for B0 was 1.81 kPa.s, and this increased to 2.08 kPa.s after crosslinking (R0). This increase can be attributed to the crosslinking effect of DCP, which strengthens the network structure within the polymer matrix, enhancing resistance to flow. The rise in viscosity at higher lignin levels reflects the increased crosslink density, as lignin acts not just as a filler but as a component that participates in the formation of a more interconnected network [37,40]. For the R10 and R20 samples, the lower η* than B10 and B20 cannot be explained.

All the polymer melt samples showed non-Newtonian shear thinning behavior (pseudoplastic). Therefore, the data for each formulation were fitted to a power–law model (Equation (2)), and a summary of the model’s parameters (*K*, *n* and R^2^) is given in Table 2. The fitted models showed generally good fits, with R^2^ > 0.9. In the blended (B0–B45) and crosslinked (R0–R45) series, *n* values generally increased with lignin content. This suggests that lignin acted as a plasticizer for PBS [41]. The *n* value for R0 was low (0.118) due to a highly crosslinked polymer network [37]. In contrast, wood–plastic composite melts show a decrease in *n* with wood content [42]. This plasticization phenomenon was also evident via a decrease in K values with increasing lignin content, except for samples R30 and R45, wherein they increased.

### 3.4. Dynamic Mechanical Analysis (DMA)

**Figure 3:**
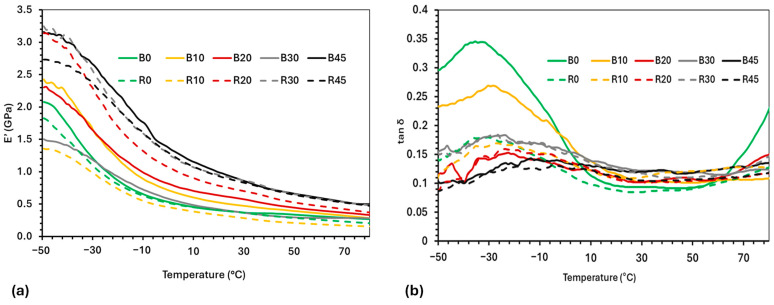
DMA thermograms of copolymers showing (**a**) storage modulus (E’) and (**b**) tan δ vs. temperature.

The viscoelastic properties (storage modulus (E′) and tan δ) of the various PBS–lignin formulations were determined by DMA to examine the influence of lignin content and crosslinking (Figure 3 and Table 3). The E′ for all the formulations decreased significantly with temperature due to passing through a glass transition phase (T_g_) around −30 °C. For the PBS–lignin blends, B45 had the highest E′ at 3.16 GPa (at −50 °C) compared to PBS at 2.08 GPa, showing that lignin effectively reinforced the polymer matrix [38,43]. Crosslinking PBS (R0) reduced its E′ by 13%; however, the addition of lignin increased E′ to 3.25 GPa (R30). These results highlight the role of DCP-induced crosslinking in enhancing the PBS and lignin network structure [28,44].

The T_g_ was determined from the tan δ max values, and was shown to increase with lignin content (Figure 3 and Table 3). For example, The T_g_ of PBS (B0) was −33 °C and increased to −13 °C at 45% lignin (B45), indicating that lignin restricts chain mobility and raises the T_g_ [38,45]. The crosslinking of PBS (R0) increased its T_g_ by 3 °C and, together with the addition of lignin, further increased its T_g_ to −4 °C (R45), demonstrating the combined effect of lignin and crosslinking on reducing molecular mobility [44,46]. It is worth mentioning that, above the T_g_ of PBS, the E′ values of the PBS–lignin formulations (B and R series) are influenced by lignin content. For example, the E′ (at 10 °C) of R0 was 0.45 GPa, and this increased to 1.05 GPa for R45. This suggests that lignin improved the stiffness of the polymer matrix due to lignin’s high T_g_ (147 °C) [47], thereby reducing chain mobility and energy dissipation [37,44].

### 3.5. Differential Scanning Calorimetry (DSC)

**Figure 4:**
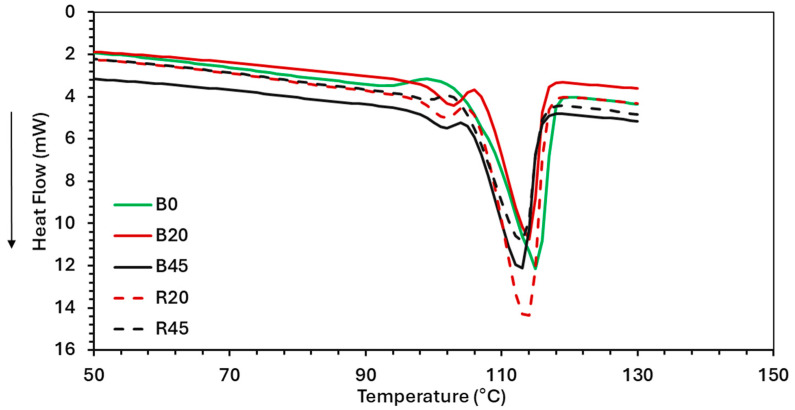
DSC thermograms (2nd heating cycle) of neat PBS and PBS–lignin copolymers.

The thermal properties (T_g_, T_c_, and T_m_) of PBS–lignin formulations were determined by DSC (Figure 4). All the PBS–lignin formulations showed a small initial T_m1_ peak around 102 °C followed by a larger main T_m2_ peak at 113–115 °C. This can be attributed to the presence of two distinct crystalline regions in the material. According to Shichao et al. [48], lignin can act as a nucleating agent, which may lead to the formation of smaller crystalline structures that melt at lower temperatures, while T_m2_ at 115 °C represents the melting of more stable, larger crystalline domains of PBS. This was also observed by Young et al., who found that the incorporation of lignin into polyester matrices can lead to changes in thermal transitions, including melting behavior [17,37]. The T_m2_ values for both R and B series ranged from 113.2 °C to 115.6 °C, indicating that the melting point remained relatively stable despite variations in lignin content and DCP cross-linking. This stability suggests that the addition of lignin does not severely disrupt the crystalline structure of PBS [49]. 

The crystallization temperature (T_c_) was obtained from the cooling curve. PBS had a T_c_ of 77 °C, and this increased with the addition of lignin (Table 4). This suggests that lignin acted as a nucleating agent. Crosslinking PBS (R0) also resulted in an increase in T_c_ by 6 °C. For the cross-linked PBS–lignin formulations, the T_c_ decreased with lignin content, suggesting that lignin restricted PBS chain mobility due to lignin’s high T_g_ of 147 °C [47], which limits PBS’s ability to reorganize into crystalline regions [49]. The crystallinity (X_c_) of the PBS–lignin B series ranged between 49 and 54%, and no obvious trends were observed (Table 4). The cross-linked PBS (R0) had an X_c_ of 44% that increased with the addition of lignin (48–50%). This suggests that lignin acted as a weak nucleating agent [29].

### 3.6. Thermogravimetric Analysis

**Figure 5:**
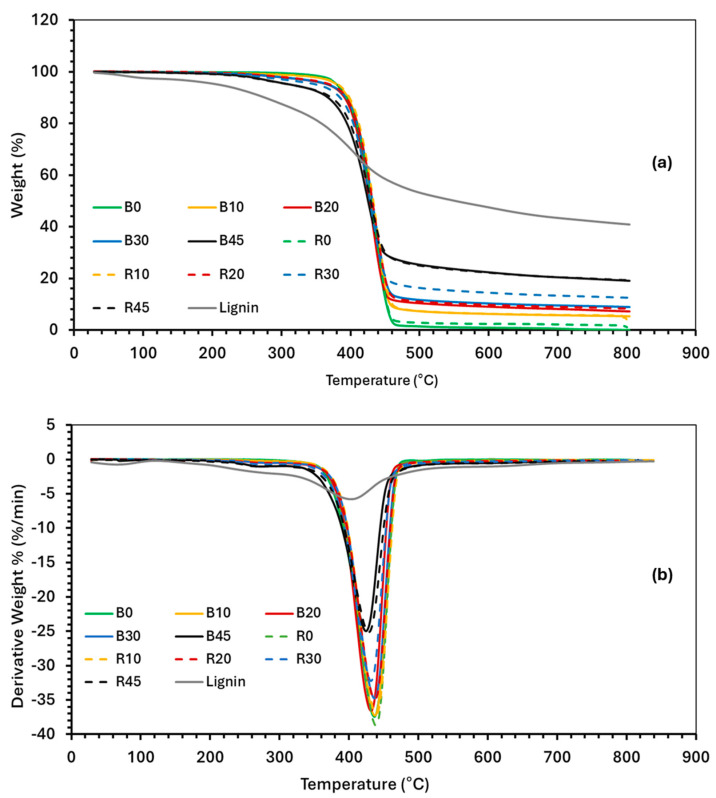
Thermograms of (**a**) thermogravimetric analysis (TGA) and (**b**) differential TGA (DTG) of PBS, lignin, and PBS–lignin copolymers.

The thermal degradation behaviors of PBS and various lignin–PBS formulations were determined by TGA and differential thermogravimetric (DTG) analysis (Figure 5). It was observed that, after the initial moisture release, the thermal degradation onset (T_onset_) of lignin started at 320 °C, which is lower than that of PBS, at 396 °C (Table 5). Cross-linking PBS (R0) did not significantly change its T_onset_. The T_onset_ for lignin was 320 °C. The T_onset_ values for the PBS–lignin formulations were comparable to PBS, suggesting that lignin did not influence PBS onset [39]. From the DTG curves, the temperatures at the peak maximum (DTG_max_) were determined (Table 5). The DTG_max_ values for lignin and PBS were 407 °C and 437 °C, respectively. For the PBS–lignin formulations, the DTG_max_ was between 426 and 437 °C. However, the intensity of the DTG_max_ peak reduced with lignin content, suggesting that lignin can be used to improve the thermal stability of PBS [29,37,50]. Lignin had a residual mass of 40% at 800 °C, and this is lower than those reported by Zhang et al. at 50% [51] and Beis et al. at 44% [52] for kraft lignin. The TGA residual material at 800 °C was shown to increase with lignin content. For example, at 45% lignin, the residual mass was around 18–19%, indicating that lignin contributes to char formation during thermal degradation in N_2_. This behavior is consistent with the findings that lignin can improve the char yield due to its aromatic structure, which provides thermal stability [37]. DCP addition was not found to have any significant effect on the thermal degradation behavior of the copolymers.

### 3.7. Mechanical Properties

The tensile properties of injection-molded PBS and PBS–lignin formulations were determined, and the results are given in Table 6. The tensile strength of PBS (B0) was 34.9 MPa and decreased to 14.5 MPa (B45) with 45% lignin. This decrease in strength trend was also observed in [29,35]. For comparison with other bioplastics, the tensile strengths of PLA, PHB, and PHBV were 64.6 [53], 22.5 and 17.0 MPa [54], respectively. Crosslinking PBS (R0) did not change its tensile strength and was shown to decrease with lignin content (R0–R45), suggesting that while DCP enhances the rigidity of the network, this did not compensate for the loss of strength associated with higher lignin content. In a study by [37], the tensile strength was improved by peroxide crosslinking; however, this depended heavily on DCP concentration. The Young’s modulus for PBS (B0) was 575 MPa, and this increased with lignin content to 1.2 GPa (B45) (Table 6). These results clearly show that lignin reinforces the PBS matrix [29,35,37]. Furthermore, the Young’s modulus values were in the range of those of PHB (1100 MPa) and PHBV (989 MPa) [54]; however, these are considerably lower than that of PLA, at 3800 MPa [53]. Crosslinking PBS (R0) increased its modulus by 39%. Furthermore, the Young’s modulus generally increased with higher lignin content. These observations are consistent with those in the literature, where the addition of unmodified lignin improved the modulus of the polymer matrix but reduced tensile strength due to phase separation, because of incompatibility between the two polymers [17,30,55].

**Figure 6:**
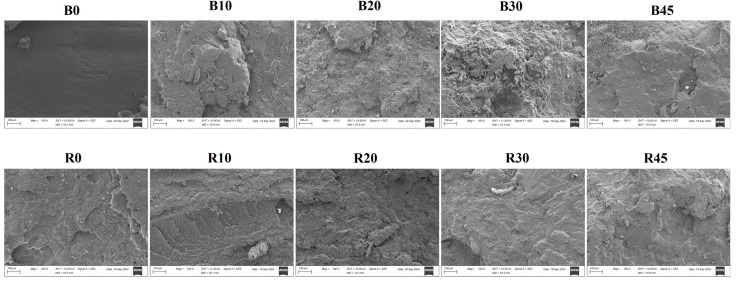
SEM micrographs showing fracture surfaces of lignin–PBS copolymers at 100× magnification.

The tensile fractured surfaces were examined by SEM in order to understand the mode of failure (Figure 6). PBS had a smooth and continuous surface typical of homogenous, ductile polymer materials, where fractures happen cohesively without significant roughness [56]. The blended PBS–lignin materials (B10, B20 and B30) showed rougher and more fragmented surfaces, indicating the poor interfacial adhesion between lignin and the PBS matrix [28,37]. As the lignin content increased (as seen in B30), the surface roughness increased, making the shapes more heterogeneous and layered. Lignin is brittle and does not mix well with PBS, which causes phase separation, making the fracture surface even rougher and more discontinuous [28,46]. Crosslinking the PBS–lignin materials (e.g., R10 and R30) produced smoother and more even surfaces compared to their blended counterparts. DCP seems to enhance the bonding between lignin and PBS, resulting in a more cohesive fracture surface with reduced visible phase separation [46]. Despite the improvement, R10, R20 and R30 still showed substantial roughness and fragmentation, suggesting that while crosslinking may have enhanced the polymer network, the inherent immiscibility of lignin and PBS is only partially mitigated. Even so, the smoother surfaces in the crosslinked samples indicate better stress distribution and improved matrix cohesion compared to the non-crosslinked blends.

The flexural properties of PBS and PBS–lignin formulations were determined, and the results are given in Table 7. The flexural strengths of PBS (B0) and crosslinked PBS (R0) were similar, around 30 MPa, and a bit lower than their tensile strengths. The flexural strength increased with 20% and 30% lignin contents in B20 and R30, respectively, which helped in resisting bending stresses and reinforcing the polymer matrix [57]. However, at higher lignin concentrations (B30, B45, and R45), the flexural strength declined. This decline could be attributed to insufficient adhesion with an excess of lignin [35,37]. The DCP crosslinked and grafted PBS–lignin (R30) showed the highest flexural strength at 41.1 MPa. Both B and R series showed an increase in flexural modulus with lignin content (for example, 0.71 GPa for B0 to 1.49 GPa for B45), indicating that a higher lignin content contributes to increased stiffness, likely due to the reinforcing effect of rigid lignin particles [29,57]. DCP-induced cross-linking did not significantly influence flexural moduli values.

## 4. Conclusions

The successful preparation of PBS–lignin copolymers demonstrated that lignin could serve as a sustainable filler/reinforcement in bioplastic production. PBS and lignin could be crosslinked and grafted to each other by reactive extrusion with DCP. Lignin enhanced stiffness and thermal stability, although it reduced tensile strength due to its limited compatibility with PBS. Crosslinking with DCP improved polymer–lignin interactions, especially at moderate lignin levels, as shown by their enhanced flexural properties and surface morphology. Based on these observations, an optimal lignin concentration appears to be around 20–30 wt. %, whereat mechanical performance and thermal stability are balanced. At higher lignin concentrations, crystallization was hindered, and phase separation occurred in non-crosslinked samples. SEM analysis confirmed improved surface integration in the crosslinked composites. While the incorporation of lignin in PBS–lignin copolymers improved properties like thermal stability and flexural modulus, this also entailed some trade-offs, such as a decrease in tensile strength. The development of PBS–lignin composites has significant potential for applications in biodegradable packaging, agricultural films, and other sustainable bioplastics, making them an attractive alternative to conventional petroleum-based plastics.

## Figures and Tables

**Table 1:** Density and gel fraction contents of PBS–lignin extruded materials.

Formulation	Density (g/cm^3^)	Gel Fraction (%)
B0	1.244	-
B10	1.256	-
B20	1.256	-
B30	1.238	-
B45	1.239	-
R0	1.248	-
R10	1.240	4.7
R20	1.262	4.3
R30	1.239	12.7
R45	1.246	26.2
Lignin	1.226	-

**Table 2:** Complex viscosity (η*) of the formulations at 1 Hz and 30 Hz and 140 °C, and the power–law fitted model equations with parameters *K* and *n*.

	η* (kPa.s)				
Formulation	1 Hz	30 Hz	K (kPa.s)	*n*	R^2^ Values
B0	1.81	0.364	1.53	0.636	0.927
B10	1.11	0.358	0.931	0.777	0.910
B20	0.573	0.279	0.509	0.867	0.896
B30	0.458	0.235	0.430	0.851	0.956
B45	0.422	0.205	0.466	0.740	0.986
R0	2.08	0.152	2.42	0.118	0.954
R10	0.989	0.282	0.840	0.737	0.947
R20	0.543	0.227	0.479	0.834	0.939
R30	0.898	0.379	0.832	0.794	0.977
R45	0.979	0.423	1.06	0.698	0.967

**Table 3:** Storage modulus, tan δ, and T_g_ of PBS–lignin copolymers.

Formulation	E′ (GPa) at −50 (°C)	E′ (GPa) at 10 (°C)	tan δ at −50 (°C)	tan δ at 10 (°C)	T_g_ (°C)
B0	2.08	0.44	0.296	0.112	−33
B10	2.43	0.59	0.233	0.137	−30
B20	2.30	0.69	0.110	0.122	−22
B30	1.51	0.47	0.154	0.138	−23
B45	3.16	1.13	0.107	0.129	−13
R0	1.82	0.45	0.144	0.097	−30
R10	1.36	0.38	0.106	0.121	−27
R20	3.14	0.88	0.089	0.124	−25
R30	3.25	1.05	0.153	0.135	−26
R45	2.73	1.05	0.087	0.123	−4

**Table 4:** Melt and crystallization behavior of neat PBS and PBS–lignin copolymers determined by DSC.

Formulation	T_m_ (°C)	T_c_ (°C)	ΔH_m_ (J/g)	X_c_ (%)
B0	115	77	58	53
B10	116	82	49	49
B20	114	82	44	50
B30	113	78	42	54
B45	114	80	29	49
R0	114	83	49	44
R10	115	80	49	50
R20	114	81	44	50
R30	114	79	39	50
R45	114	76	29	48
100% Crystalline PBS			110.3	

**Table 5:** Thermogravimetric data for neat PBS, lignin, and PBS–lignin copolymers.

Formulation	T_onset_ (°C)	Residue at 800 °C (%)	DTG_max_ (°C)
B0	396 (±6.8)	0 (±0)	437 (±0.6)
B10	397 (±5.4)	6 (±0.5)	434 (±5.0)
B20	398 (±2.1)	10 (±2.0)	429 (±6.0)
B30	397 (±1.3)	10 (±1.4)	436 (±0.6)
B45	385 (±1.5)	18 (±0.8)	426 (±1.0)
R0	397 (±2.1)	0 (±0.2)	440 (±1.8)
R10	399 (±6.5)	6 (±0.7)	437 (±3.5)
R20	396 (±7.3)	9 (±0.5)	433 (±5.6)
R30	399 (±1.4)	13 (±0.6)	430 (±5.5)
R45	396 (±1.1)	19 (±0.7)	429 (±1.1)
Lignin	320 (±1.5)	40 (±0.2)	407 (±1.9)

**Table 6:** Tensile test values for neat PBS and PBS–lignin copolymers.

Formulation	Tensile Strength (MPa)	Young’s Modulus (MPa)
B0	34.9 ± 0.9 ^a^	575 ± 65 ^c^
B10	27.9 ± 1.9 ^b^	922 ± 77 ^b^
B20	22.2 ± 1.7 ^c^	960 ± 77 ^b^
B30	17.7 ± 1.6 ^d^	969 ± 77 ^b^
B45	14.5 ± 0.8 ^e^	1206 ± 132 ^a^
R0	35.9 ± 2.1 ^a^	801 ± 121 ^c’^
R10	26.3 ± 2.8 ^b^	751 ± 40 ^b’^
R20	18.9 ± 0.9 ^c^	716 ± 49 ^b’^
R30	17.2 ± 0.4 ^c,d^	910 ± 85 ^b^
R45	14.1 ± 1.7 ^d,e^	1256 ± 162 ^a^

^a^ Values are mean ± standard deviation. Where necessary, the standard deviation has been rounded up to the mean’s reported precision. ^b^ Statistical differences in the results were measured via Tukey test (*p*-value < 0.05) and are shown by superscript letters (a–e,b’,c’).

**Table 7:** Flexural test values for neat PBS and PBS–lignin copolymers.

Formulation	Flexural Strength (MPa)	Flexural Modulus (MPa)
B0	29.9 ± 3.2 ^a^	705 ± 89 ^a^
B10	35.2 ± 2.8 ^b^	906 ±114 ^b^
B20	37.3 ± 2.5 ^c^	1025 ±93 ^b,c^
B30	32.6 ± 1.8 ^a,b^	1125 ± 50 ^c^
B45	30.4 ± 2.9 ^a^	1486 ± 150 ^d^
R0	30.7 ± 2.6 ^a^	727 ± 84 ^a^
R10	34.6 ± 3.7 ^a,b^	864 ± 104 ^a,b^
R20	36.2 ± 3.9 ^b,c^	961 ±135 ^b^
R30	41.1 ± 5.3 ^c^	1213 ± 167 ^c^
R45	29.5 ± 2.5 ^a^	1482 ± 228 ^d^

^a^ Values are mean ± standard deviation. Where necessary, the standard deviation has been rounded up to the mean’s reported precision. ^b^ Statistical differences in the results were measured via Tukey test (*p*-value < 0.05) and are shown by superscript letters (a–d).

## Data Availability

The data presented in this study are available on request from the corresponding author.
